# Supplementary material for: GerPaCyst - The trial protocol of the prospective, multicenter, interdisciplinary German Pancreas Club Cyst Registry
Source: PLoS One. 2025 Nov 25;20(11):e0335809. doi: 10.1371/journal.pone.0335809 (PMC12646447; doi:10.1371/journal.pone.0335809)
Supplement: S1 Table — (DOCX) [file pone.0335809.s001.docx]

| **Variable / Field Name** |
| --- |
| **Study_Id** |
| **Today** |
| **Date_Of_Birth** |
| **Hospital_Id** |
| **Gender** |
| **Comments** |
| **Date_First_Visit** |
| **Death_Date** |
| **End_Other** |
| **Surgery_For_Suspected_Malignancy** |
| **Staging** |
| **Date_Of_Operation** |
| **Type_Of_Operation** |
| **Multivisceral_Resection** |
| **Op_Method** |
| **Conversion_To_Open_Surgery** |
| **Clavien_Dindo** |
| **Postoperative_Fistula** |
| **Postoperative_Pneumonia** |
| **Pulmonary_Artery_Embolism** |
| **Postoperative_Myocardial_Infarction** |
| **Postoeprative_Apoplex** |
| **Postpancreatectomy_Hemmorhage** |
| **Hospital_Stay** |
| **Hospital_Mortality** |
| **Date_Death** |
| **Days_Surgery_Death** |
| **Day_Mortality** |
| **Surgical_Pathology** |
| **Md_Ipmn** |
| **Bd_Ipmn** |
| **M_Ipmn** |
| **Concurrent** |
| **Seperated** |
| **Specify_Seperated** |
| **Sca** |
| **Diagnosis** |
| **Type_Of_Dysplasia** |
| **Location_Of_Tumor** |
| **T** |
| **N** |
| **Positive_Lymphnodes** |
| **Total_Number_Of_Lymph_Nodes** |
| **Metastasis** |
| **Perineural_Invasion** |
| **Lymphangiosis** |
| **Microvessel_Invasion** |
| **Circumferential_Resection_Margin** |
| **Residual_Tumor** |
| **Relapse** |
| **Date_Relapse** |
| **Location_Relapse** |
| **Specify_Other_Relapse** |
| **Body_Height** |
| **Body_Weight** |
| **Malignant_Diseases** |
| **Patients_History_Malignancies** |
| **Patient_History_Other** |
| **History_Pdac** |
| **Degree_Relative** |
| **Age_At_Diagnosis** |
| **Cardiovascular_Disease** |
| **Coronary_Heart_Disease** |
| **Heart_Insufficiency** |
| **Nyha** |
| **Cerebrovascular_Event** |
| **Dialysis** |
| **Copd** |
| **Pavk** |
| **Metabolic_Disease** |
| **Diabetes** |
| **New_Onset_Diabetes** |
| **Date_New_Diabetes** |
| **Year_Of_Diagnosis** |
| **Diabetes_Type** |
| **Onset_Insulin_Dependence** |
| **Exocrine_Pancreatic** |
| **Elastase** |
| **Substitution** |
| **Risk_Factors** |
| **Pack_Years** |
| **Risk_Factor_Others** |
| **Symptoms** |
| **Tumor_Related_Jaundice** |
| **Jaundice_Absolute_Indication** |
| **Pancreatitis_Ipmn** |
| **Pancreatitis_Relative** |
| **Symptoms_Other** |
| **Fit_For_Surgery** |
| **Ecog** |
| **Asa_Score** |
| **Eq-5** |
| **Vas** |
| **Presumed_Diagnosis** |
| **Cyst_Discovery** |
| **Surveillance_Indication** |
| **Year_First_Diagnosis** |
| **Planned_Treatment** |
| **Imaging_Type** |
| **Eus** |
| **Ct** |
| **Type_Mri** |
| **T2** |
| **Mrcp** |
| **Dwi** |
| **Mri_Other** |
| **Number_Of_Cysts** |
| **Largest_Cyst** |
| **Mpd_Diameter** |
| **Head** |
| **Neck_Mm** |
| **Body** |
| **Tail** |
| **Change_In_Caliber** |
| **Distal_Pancreatic_Atrophy** |
| **Common_Bile_Duct_Dilatation** |
| **Suspicious_Findings** |
| **Other_Suspicious_Findings** |
| **Serum_Ca19_9** |
| **Relative_Indication_Ca19_9** |
| **Hba1c** |
| **Cyst_Fluid Analysis Results** |
| **Molecular_Analysis** |
| **Cyst_Cytology** |
| **Cyst_Cytology_Yes** |
| **Cytology_Absolute_Indication** |
| **Edta** |
| **Serum** |
| **Biobanking** |
| **Liquid_Biopsy**  **Number Of Cysts** |
| **Cyst_Morphology** |
| **Imaging_C1** |
| **Location_File** |
| **C1_Location** |
| **C1_Diameter** |
| **C1_Diameter_Relative** |
| **C1pattern** |
| **C1_Growth** |
| **C1_Thickened_Wall** |
| **C1_Cyst_Wall** |
| **C1_Enhanced** |
| **C1_Mural_Nodules** |
| **C1_Mural_Indication** |
| **C1_Connection_Mpd** |
| **C1_Solid_Components** |
| **C1_Calcifications** |
| **C1_Shear_Wave** |
| **C1_Shear_Wave_Elastography** |
|  |
