# Supplementary material for: GerPaCyst - The trial protocol of the prospective, multicenter, interdisciplinary German Pancreas Club Cyst Registry
Source: PLoS One. 2025 Nov 25;20(11):e0335809. doi: 10.1371/journal.pone.0335809 (PMC12646447; doi:10.1371/journal.pone.0335809)
Supplement: S4 Table — (DOCX) [file pone.0335809.s004.docx]

**Blood and Cross Sectional Imaging parameters for pancreatic cysts**

1. **NGS Panel**

| ABL1 | Resistance gene / AS Cancer Hotspot Panel and SureSelectCancerAIO |
| --- | --- |
| AKT1 | Custom panel Agilent, ThermoFisher |
| ALK | nNGM |
| AMER1 | UKSH |
| APC | UKSH |
| ARID1A | Gene list |
| ASXL1 | CRC MF IKMB suggestion |
| ATM | UKSH |
| AXL | Overlap EXTARGET |
| BCL2 | Overlap EXTARGET |
| BRAF | Original NGS panel |
| BRCA1 | Overlap EXTARGET |
| BRCA2 | CRC MF IKMB suggestion |
| CCND1 | Driver mutation MM, CRC panel |
| CD274 | Custom panel Agilent, ThermoFisher |
| CD79B | UKSH |
| CDH1 | Resistance gene / AS Cancer Hotspot Panel and SureSelectCancerAIO |
| CDK4 | Resistance gene / AS Cancer Hotspot Panel and SureSelectCancerAIO |
| CDK6 | Resistance gene / AS Cancer Hotspot Panel and SureSelectCancerAIO |
| CDKN2A | Original NGS panel |
| CHEK2 | CHIP genes (PMID: 34298011) |
| CSF1R | Resistance gene / AS Cancer Hotspot Panel and SureSelectCancerAIO |
| CTNNB1 | nNGM |
| DIS3 | Driver mutation MM |
| DNMT3A | CHIP genes (PMID: 34298011) |
| DUSP2 | Driver mutation MM |
| EGFR | Original NGS panel |
| EGR1 | Driver mutation MM |
| ERBB2 | Original NGS panel |
| ERBB3 | Driver gene COR, CRC panel |
| ERBB4 | Resistance gene / AS Cancer Hotspot Panel and SureSelectCancerAIO |
| EZH2 | UKSH internal suggestion |
| FAM46C (TENT5C) | Driver mutation MM |
| FBXW7 | UKSH |
| FGFR1 | nNGM |
| FGFR2 | nNGM |
| FGFR3 | nNGM |
| FGFR4 | nNGM |
| FLT3 | Overlap EXTARGET |
| GNA11 | UKSH |
| GNAQ | UKSH |
| GNAS | Resistance gene / AS Cancer Hotspot Panel and SureSelectCancerAIO |
| HIST1H1E | Driver mutation MM |
| HRAS | Original NGS panel |
| IDH1 | nNGM and UKSH internal suggestion |
| IDH2 | nNGM |
| IGLL5 | Driver mutation MM |
| JAK2 | Resistance gene / AS Cancer Hotspot Panel and SureSelectCancerAIO |
| JAK3 | Resistance gene / AS Cancer Hotspot Panel and SureSelectCancerAIO |
| KDR | Resistance gene / AS Cancer Hotspot Panel and SureSelectCancerAIO |
| KEAP1 | Original NGS panel |
| KIT | Original NGS panel |
| KLHL6 | Driver mutation MM |
| KMT2C | UKSH |
| KMT2D | UKSH |
| KRAS | Original NGS panel |
| LRP1B | CRC MF IKMB suggestion |
| LTB | Driver mutation MM |
| MAP2K1 | nNGM |
| MAP2K2 | Overlap EXTARGET |
| MAPK1 | Overlap EXTARGET |
| MAPK3 | Overlap EXTARGET |
| MDM2 | Resistance gene / AS Cancer Hotspot Panel and SureSelectCancerAIO |
| MET | Original NGS panel |
| MLH1 | Resistance gene / AS Cancer Hotspot Panel and SureSelectCancerAIO |
| MTOR | Driver gene COR, CRC panel |
| MYC | Resistance gene / AS Cancer Hotspot Panel and SureSelectCancerAIO |
| MYCN | Resistance gene / AS Cancer Hotspot Panel and SureSelectCancerAIO |
| MYD88 | Custom panel Agilent and UKSH internal suggestion |
| NF1 | CHIP genes (PMID: 34298011) |
| NFE2L2 | Original NGS panel |
| NOTCH1 | CRC MF IKMB suggestion |
| NOTCH2 | UKSH |
| NRAS | Original NGS panel |
| NTRK1 | nNGM |
| NTRK2 | nNGM |
| NTRK3 | UKSH internal suggestion |
| PARP1 | Overlap EXTARGET |
| PDGFRA | Original NGS panel |
| PDGFRB | Resistance gene / AS Cancer Hotspot Panel and SureSelectCancerAIO |
| PIK3CA | Original NGS panel |
| PIK3R1 | Custom panel Agilent, ThermoFisher |
| PPM1D | CHIP genes (PMID: 34298011) |
| PRKD2 | Driver mutation MM |
| PTEN | Original NGS panel |
| PTPN11 | Driver mutation MM, Driver gene COR |
| RB1 | Driver mutation MM |
| RET | nNGM |
| RNF43 | UKSH |
| ROS1 | nNGM |
| SDHA | Original NGS panel |
| SF3B1 | Original NGS panel |
| SMAD4 | Original NGS panel |
| SMARCB1 | Resistance gene / AS Cancer Hotspot Panel and SureSelectCancerAIO |
| SMO | Resistance gene / AS Cancer Hotspot Panel and SureSelectCancerAIO |
| SOX9 | CRC MF IKMB suggestion |
| SRC | Resistance gene / AS Cancer Hotspot Panel and SureSelectCancerAIO |
| SRSF2 | CHIP genes (PMID: 34298011) |
| STAT3 | CHIP genes (PMID: 34298011) |
| STK11 | nNGM |
| TCF7L2 | UKSH |
| TET2 | CHIP genes (PMID: 34298011) |
| TGFBR2 | CRC MF IKMB suggestion |
| TP53 | Original NGS panel |
| TSC1 | Resistance gene / AS Cancer Hotspot Panel and SureSelectCancerAIO |
| TSC2 | Resistance gene / AS Cancer Hotspot Panel and SureSelectCancerAIO |
| UGT1A1 | Custom panel Roche, ThermoFisher |
| VHL | Resistance gene / AS Cancer Hotspot Panel and SureSelectCancerAIO |
| WT1 | Resistance gene / AS Cancer Hotspot Panel and SureSelectCancerAIO |
| ALK | Most common intron/exon of most common fusion genes (PMID: 26437441) |
| BCR | Most common intron/exon of most common fusion genes (PMID: 26437441) |
| BCR | Most common intron/exon of most common fusion genes (PMID: 26437441) |
| BRAF | Most common intron/exon of most common fusion genes (PMID: 26437441) |
| FGFR2 | Most common intron/exon of most common fusion genes (PMID: 26437441) |
| FGFR2 | Most common intron/exon of most common fusion genes (PMID: 26437441) |
| FGFR3 | Most common intron/exon of most common fusion genes (PMID: 26437441) |
| NTRK1 | Most common intron/exon of most common fusion genes (PMID: 26437441) |
| NTRK1 | Most common intron/exon of most common fusion genes (PMID: 26437441) |
| NTRK1 | Most common intron/exon of most common fusion genes (PMID: 26437441) |
| NTRK1 | Most common intron/exon of most common fusion genes (PMID: 26437441) |
| NTRK1 | Most common intron/exon of most common fusion genes (PMID: 26437441) |
| RET | Most common intron/exon of most common fusion genes (PMID: 26437441) |
| ROS1 | Most common intron/exon of most common fusion genes (PMID: 26437441) |
| rs1065457 | Pengelly genotyping SNP |
| rs1200349 | Pengelly genotyping SNP |
| rs12102203 | Pengelly genotyping SNP |
| rs1344 | Pengelly genotyping SNP |
| rs140679 | Pengelly genotyping SNP |
| rs1657502 | Pengelly genotyping SNP |
| rs17548783 | Pengelly genotyping SNP |
| rs2032653 | Pengelly genotyping SNP |
| rs2273171 | Pengelly genotyping SNP |
| rs2301771 | Pengelly genotyping SNP |
| rs2889732 | Pengelly genotyping SNP |
| rs34581739 | Pengelly genotyping SNP |
| rs4478844 | Pengelly genotyping SNP |
| rs4664475 | Pengelly genotyping SNP |
| rs495680 | Pengelly genotyping SNP |
| rs7653897 | Pengelly genotyping SNP |
| rs8473 | Pengelly genotyping SNP |

1. **Cross-Sectional Imaging Parameters for Pancreatic Cyst Evaluation**

**High-Risk Stigmata (Absolute Indications for Surgery)**

**Morphological Features**

- **Enhancing solid component within cyst**
  - Contrast-enhancing mural nodule >5 mm
  - Solid mass within or adjacent to cyst
  - Irregular enhancing tissue projections

**Ductal Features**

- **Main pancreatic duct diameter ≥10 mm**
  - Measured at widest point
  - Associated with main duct IPMN

**Clinical Features**

- **Obstructive jaundice**
  - In patient with cystic lesion of pancreatic head
  - Bile duct dilatation with associated pancreatic cyst

**Worrisome Features (Indications for EUS or Closer Surveillance)**

**Size Parameters**

- **Cyst diameter ≥3 cm**
  - Measured in largest dimension
  - Associated with increased malignancy risk

**Wall Characteristics**

- **Thickened cyst walls**
  - Wall thickness >2-3 mm
  - Irregular wall contour
- **Enhancing cyst walls**
  - Enhancement on post-contrast imaging
  - Suggests inflammation or malignancy

**Ductal Parameters**

- **Main pancreatic duct diameter 5-9 mm**
  - Intermediate dilatation
  - May indicate mixed-type IPMN
- **Abrupt change in pancreatic duct caliber**
  - Sudden narrowing or dilatation
  - Associated with distal pancreatic atrophy

**Internal Features**

- **Non-enhancing mural nodules**
  - Internal projections without enhancement
  - Size >5 mm considered significant

**Associated Findings**

- **Lymphadenopathy**
  - Regional lymph node enlargement
  - May suggest malignant transformation

**Additional Morphological Characteristics**

**Cyst Classification by Location**

- **Main duct type**
  - Dilatation of main pancreatic duct >5 mm
  - Segmental or diffuse involvement
- **Branch duct type**
  - Cystic dilatation of branch ducts
  - "Grape-like" or "honeycomb" appearance
- **Mixed type**
  - Features of both main and branch duct involvement

**Communication Patterns**

- **Duct communication**
  - Direct connection to main pancreatic duct
  - Visualized on MRCP or ERCP
- **No duct communication**
  - Isolated cystic lesion
  - May suggest mucinous cystadenoma

**Internal Architecture**

- **Septations**
  - Number and thickness of internal septa
  - Enhancement pattern of septations
- **Internal debris/mucin**
  - High T1 signal on MRI
  - Dependent layering
  - "Mucin plug" sign
- **Hemorrhage**
  - Blood products within cyst
  - Various signal intensities on MRI

**Multiplicity**

- **Single cyst**
  - Isolated lesion
- **Multiple cysts**
  - Multifocal disease
  - Suggests familial predisposition or field defect

**Parenchymal Changes**

**Pancreatic Atrophy**

- **Focal atrophy**
  - Localized parenchymal loss
  - Associated with ductal obstruction
- **Diffuse atrophy**
  - Generalized parenchymal thinning
  - May indicate chronic pancreatitis

**Enhancement Patterns**

- **Parenchymal enhancement**
  - Normal vs. decreased enhancement
  - Perfusion abnormalities
- **Peripancreatic changes**
  - Fat stranding
  - Vascular involvement

**Vascular Involvement**

**Arterial Relationships**

- **Superior mesenteric artery**
- **Celiac axis**
- **Splenic artery**
- **Gastroduodenal artery**

**Venous Relationships**

- **Portal vein**
- **Superior mesenteric vein**
- **Splenic vein**

**Imaging Protocol Requirements**

**CT Pancreatic Protocol**

- **Pre-contrast phase**
- **Pancreatic parenchymal phase** (40-50 seconds)
- **Portal venous phase** (70-80 seconds)
- **Delayed phase** (3-5 minutes) - optional
- **Thin section reconstruction** (≤3 mm)

**MRI/MRCP Protocol**

- **T1-weighted imaging**
  - Pre and post-contrast
  - Fat-suppressed sequences
- **T2-weighted imaging**
  - Heavily T2-weighted for MRCP
  - Axial and coronal planes
- **Diffusion-weighted imaging** (DWI)
- **Dynamic contrast enhancement**

**EUS Complementary Features**

- **Wall thickness measurement**
- **Mural nodule characterization**
- **Septation analysis**
- **Doppler flow assessment**
- **Fine needle aspiration capability**

**Risk Stratification Categories**

**Low Risk**

- Branch duct IPMN <3 cm
- No worrisome features
- No high-risk stigmata

**Intermediate Risk**

- Presence of worrisome features
- No high-risk stigmata
- Requires
