# Supplementary material for: GerPaCyst - The trial protocol of the prospective, multicenter, interdisciplinary German Pancreas Club Cyst Registry
Source: PLoS One. 2025 Nov 25;20(11):e0335809. doi: 10.1371/journal.pone.0335809 (PMC12646447; doi:10.1371/journal.pone.0335809)
Supplement: Attachment — Submitted filename: kch edit Rebutal Letter GERPACYST.pdf [file pone.0335809.s005.pdf]

UNIVERSITÄTSKLINIKUM
Schleswig-Holstein

UKSH, Klinik für Chirurgie, Ratzeburger Allee 160, 23538 Lübeck

Editor-in Chief Prof. Emily Chenette and
Prof. George Vousden
PLOS One

Campus Lübeck

Klinik für Chirurgie

Direktor: Prof. Dr. med. Tobias Keck

PD Dr. med. Kim C. Honselmann

Tel.: 0451 500-75790, **Fax:** -40194
E-Mail: kim.honselmann@uksh.de
www.uksh.de

DGAV Exzellenzzentrum Minimal Invasive Chirurgie
Universitäres Gefäßzentrum
DKG Krebsgesellschaft Zertifiziertes Onkologisches Zentrum
DKG Krebsgesellschaft Zertifiziertes Pankreaskarzinom Zentrum
DKG Krebsgesellschaft Zertifiziertes Darmkrebszentrum

Datum: September 17th, 2025

RE: Resubmission of our Manuscript: "GerPaCyst- The Trial Protocol Of The Prospective, Multicenter, Interdisciplinary German Pancreas Club" (PONE-D-25-34705)

Dear Prof. Chenette and Prof. Vousden,
Dear members of the Editorial Board,

Thank you for giving us the opportunity to resubmit our manuscript entitled: **"GerPaCyst- The Trial Protocol Of The Prospective, Multicenter, Interdisciplinary German Pancreas Club".**

We have answered the reviewer's comments to the best of our knowledge and hope you find them satisfactory. The manuscript has been uploaded with tracked changes and all changes highlighted in yellow.

Sincerely,

Kim C. Honselmann
Ass. Prof. of Surgery
Luebeck, Germany

UKSH Wissen schafft Gesundheit

Editorial comments:

1. "Please ensure that your manuscript meets PLOS ONE's style requirements, including those for file naming. The PLOS ONE style templates can be found at

https://journals.plos.org/plosone/s/file?id=wjVg/PLOSOne_formatting_sample_main_body.pdf and

https://journals.plos.org/plosone/s/file?id=ba62/PLOSOne_formatting_sample_title_authors_affiliations.pdf"

Answer 1: We changed the manuscript according to the guidelines and have highlighted those changes in yellow throughout the manuscript.

2." Thank you for stating the following financial disclosure: [Parts of the study are curreny funded by The Federal Ministry of Education and Research (BMBF) (Funding number: 01KD2412A) and was supported by the Clinician-scientist school Luebeck (project number 413535489) to KCH.]. "

"Please state what role the funders took in the study. If the funders had no role, please state: ""The funders had no role in study design, data collection and analysis, decision to publish, or preparation of the manuscript.""

"If this statement is not correct you must amend it as needed. "

Answer 2: We amended the statement as follows: *"The funders had no role in study design, data collection and analysis, decision to publish, or preparation of the manuscript."" Page 18, line 407*

3." When completing the data availability statement of the submission form, you indicated that you will make your data available on acceptance. We strongly recommend all authors decide on a data sharing plan before acceptance, as the process can be lengthy and hold up publication timelines. Please note that, though access restrictions are acceptable now, your entire data will need to be made freely accessible if your manuscript is accepted for publication. This policy applies to all data except where public deposition would breach compliance with the protocol approved by your research ethics board. If you are unable to adhere to our open data policy, please kindly revise your statement to explain your reasoning and we will seek the editor's input on an exemption. Please be assured that, once you have provided your new statement, the assessment of your exemption will not hold up the peer review process."

Answer 3: We thank the reviewers for this comment. As this is a mere study protocol, there is no data available currently as the study is still in its data acquisition phase. As our study continues, we will make the outcomes available through publications and make sure to initiate a data sharing plan now.

4. "Please amend your authorship list in your manuscript file to include authors Dr. Matthias Birth, Louisa Bolm, Ruediger Braun, Ihsan Ekin Demir, Maximilian Denzinger, Beate Drews, Ana Dugic, Thomas Ewers, Josephine-Alisa Grandi, Susanne Haberer, Christoph Ammer-Hermenau, Philip Hildebrandt, Felix Huettner, Katja Janke, Katja Kilani, Georg Lamprecht, Jens Schuette, Jolina Michaelis, Albrecht Neesse, Rene Wilke, Lukas Perkhofer, Sebastian Rasch, Maximilian Reichert, Martina Mueller-Schilling, and Thorben Sauer."

Answer 4: We have amended the authorship list accordingly line 4-14.

UKSH Wissen schafft Gesundheit

5. One of the noted authors is a group or consortium [the GerPaCyst Study Group]. In addition to naming the author group, please list the individual authors and affiliations within this group in the acknowledgments section of your manuscript. Please also indicate clearly a lead author for this group along with a contact email address.

Answer 5: Thank you for this comment. We have adjusted the listing as requested and have added the necessary information about the Study Group in the acknowledgments line 418ff.

6. We notice that your supplementary tables are included in the manuscript file. Please remove them and upload them with the file type 'Supporting Information'. Please ensure that each Supporting Information file has a legend listed in the manuscript after the references list.

Answer 6: Supplementary tables have been removed and added as supporting information. Legends have been added after the references list as “Supporting Information”.

7. Please include captions for your Supporting Information files at the end of your manuscript, and update any in-text citations to match accordingly. Please see our Supporting Information guidelines for more information: http://journals.plos.org/plosone/s/supporting-information.

Answer 7: We have modified the manuscript according to the recommendations line 598ff.

8. If the reviewer comments include a recommendation to cite specific previously published works, please review and evaluate these publications to determine whether they are relevant and should be cited. There is no requirement to cite these works unless the editor has **indicated otherwise.**

Answer 8: Thank you for this statement. None of the reviewers have done so.

9. Please review your reference list to ensure that it is complete and correct. If you have cited papers that have been retracted, please include the rationale for doing so in the manuscript text, or remove these references and replace them with relevant current references. Any changes to the reference list should be mentioned in the rebuttal letter that accompanies your revised manuscript. If you need to cite a retracted article, indicate the article's retracted status in the References list and also include a citation and full reference for the retraction notice.

Answer 9: We have checked our references accordingly and did not cite retracted papers.

Reviewers' comments:

Reviewer's Responses to Questions

Comments to the Author

1. Does the manuscript provide a valid rationale for the proposed study, with clearly identified and justified research questions?

The research question outlined is expected to address a valid academic problem or topic and contribute to the base of knowledge in the field.

Reviewer #1: Yes

Reviewer #2: Yes

---

2. Is the protocol technically sound and planned in a manner that will lead to a meaningful outcome and allow testing the stated hypotheses?

The manuscript should describe the methods in sufficient detail to prevent undisclosed flexibility in the experimental procedure or analysis pipeline, including sufficient outcome-neutral conditions (e.g. necessary controls, absence of floor or ceiling effects) to test the proposed hypotheses and a statistical power analysis where applicable. As there may be aspects of the methodology and analysis which can only be refined once the work is undertaken, authors should outline potential assumptions and explicitly describe what aspects of the proposed analyses, if any, are exploratory.

Reviewer #1: Partly

Reviewer #2: Yes

Answer 2: For Reviewer #1: Since this is not an interventional trial, controls are not described. We have added methodology for building a multi-omics risk stratification model in line 275ff.

---

3. Is the methodology feasible and described in sufficient detail to allow the work to be replicable?

Descriptions of methods and materials in the protocol should be reported in sufficient detail for another researcher to reproduce all experiments and analyses. The protocol should describe the appropriate controls, sample size calculations, and replication needed to ensure that the data are robust and reproducible.

Reviewer #1: No

Reviewer #2: Yes

Answer 3: Reviewer#1: We see your concerns. As this is a registry trial, the research questions in the future are not sufficiently foreseeable at this point. However, our primary goal is to generate a multi-omics model for risk stratification of pancreatic cyst progression. For this, we added the detailed methodology and hope to answer your question sufficiently (Line 282ff.

---

4. Have the authors described where all data underlying the findings will be made available when the study is complete?

The PLOS Data policy requires authors to make all data underlying the findings described in their manuscript fully available without restriction, with rare exception, at the time of publication. The data should be provided as part of the manuscript or its supporting information or deposited to a public repository. For example, in addition to summary statistics, the data points behind means, medians and variance measures should be available. If there are restrictions on publicly sharing data—e.g. participant privacy or use of data from a third party—those must be specified.

Reviewer #1: No

Reviewer #2: Yes

Answer 4: We thank Reviewer #1 and #2 for this comment. As we have described and outlined above, this is a mere study protocol. There is no data available currently as the study is still in its data acquisition phase. There are no concrete data points to be shared at this moment. As our study continues, we will make the outcomes available through publications. We have added a comment regarding publication of our findings in the manuscript line 354f: "Participant-level dataset and statistical codes can be obtained from KCH."

---

5. Is the manuscript presented in an intelligible fashion and written in standard English?

PLOS ONE does not copyedit accepted manuscripts, so the language in submitted articles must be clear, correct, and unambiguous. Any typographical or grammatical errors should be corrected at revision, so please note any specific errors here.

Reviewer #1: Yes

Reviewer #2: Yes

Answer 5: We thank Reviewer #1 and #2 for their comment.

---

6. Review Comments to the Author

(Please upload your review as an attachment if it exceeds 20,000 characters)

Reviewer #1: This is the protocol paper of the prospective, multicenter German Pancreas Club cyst registry.
Minor Comments:
1. Clarification of presumed diagnosis criteria:
The protocol mentions inclusion of patients with a "presumed diagnosis". However, it is unclear what specific clinical, imaging, or laboratory criteria are used to define this presumed diagnosis. Please clarify the diagnostic criteria and specify how interobserver variation is addressed in such assessments. This information should be explicitly described in the protocol.

Answer 1: We added our criteria for diagnosis of the different pancreatic cysts in the manuscript under "Participants, Eligibility criteria and outcomes" line 199ff.

2. Handling and evaluation of imaging data:
It is not clear whether the imaging findings are assessed solely based on site-reported entries in the case report forms or whether a central image review is planned. If central review of imaging data is not conducted, this should be explicitly stated. In that case, please describe how consistency across institutions (e.g., interobserver variability) will be managed or minimized. This point should be clearly addressed in the study protocol.

Answer 2: We thank Reviewer #1 for this comment. We will perform central image review. We have addressed this in line 205ff.

Reviewer #2: Comment
This is a large observational study of the long-term follow-up of pancreatic cystic lesions in Germany. Prospective studies of pancreatic cystic lesions are limited and the findings from this study are expected to be important. Some comments are provided below.

1. Abstract lacks primary endpoints, making it difficult to understand the study summary.
2. Is there a defined objective number of cases?
3. The evaluation items are difficult to understand. For example, the items to be evaluated in blood tests and in imaging tests. Why not list them all in a table?

Answer: We have addressed 1) in the abstract and have added the necessary information in line 86ff. Regarding 2) we have adjusted the manuscript accordingly in line 434f . Regarding comment 3) we have added a table to the supporting information addressing the items that are being evaluated at the moment. (S4 Table). However, this might change over the course of the 20 year study period.

---

7. PLOS authors have the option to publish the peer review history of their article (what does this mean?). If published, this will include your full peer review and any attached files.

Answer 7: Yes, please include the peer review.
